# The Association Between Amniocentesis and Adverse Pregnancy Outcomes in Pregnancies with Normal/Reportable Test Results: An Indication-Based Comparison with Non-Invasive Prenatal Testing

**DOI:** 10.3390/diagnostics16060867

**Published:** 2026-03-14

**Authors:** Burak Bayraktar, Hakan Golbasi, Melda Kuyucu, Ceren Golbasi, Ibrahim Omeroglu, Kaan Okan Alkan, Sevim Tuncer Can, Miyase Gizem Bayraktar, Atalay Ekin

**Affiliations:** 1 Department of Perinatology, Turgut Ozal University Malatya Training and Research Hospital, Malatya 44900, Turkey; 2 Department of Perinatology, University of Health Sciences Ankara Etlik City Hospital, Ankara 06170, Turkey; 3 Department of Obstetrics and Gynecology, University of Health Sciences Tepecik Training and Research Hospital, Izmir 35020, Turkey; cerengolbasi@gmail.com (C.G.); alkankaanokan@gmail.com (K.O.A.); drmiyasegizem@gmail.com (M.G.B.); 4 Department of Perinatology, University of Health Sciences Tepecik Training and Research Hospital, Izmir 35020, Turkey; drhkngolbasi@gmail.com (H.G.); melda_kuyucu@hotmail.com (M.K.); dribrahimomeroglu@gmail.com (I.O.); drsevimtuncer@hotmail.com (S.T.C.); atalayekin@hotmail.com (A.E.); 5 Department of Perinatology, University of Health Sciences Izmir City Hospital, Izmir 35540, Turkey

**Keywords:** amniocentesis, non-invasive prenatal testing, fetal loss, preterm birth, birth weight, perinatal outcomes

## Abstract

**Background/Objectives**: To compare the maternal, fetal, and neonatal outcomes of pregnancies undergoing amniocentesis with those undergoing non-invasive prenatal testing (NIPT), within a cohort of women with comparable clinical indications, aiming to evaluate differences in adverse outcomes in a risk-indicated population. **Methods**: In this retrospective cohort study, pregnancy outcomes of 2044 pregnant women who underwent amniocentesis and 7668 pregnant women who underwent NIPT were evaluated using single-center data. The analysis was restricted to pregnancies with normal/reportable test results and without structural or genetic anomalies. Pregnancy loss outcomes were evaluated in the full cohort, while perinatal outcomes were analyzed among cases with available delivery data (377 amniocentesis and 2063 NIPT cases). Pregnancy and perinatal outcomes, including miscarriage, intrauterine fetal demise (IUD), preterm birth (PTB), pregnancy-induced hypertensive diseases (PIHDs), gestational diabetes mellitus (GDM), intrahepatic cholestasis of pregnancy (ICP), low birth weight (LBW), small for gestational age (SGA), and low APGAR scores (<7), were evaluated. Multivariate logistic regression analysis was performed to adjust for potential confounding factors, and adjusted odds ratios (aORs) with 95% confidence intervals (CIs) were reported. **Results**: Amniocentesis was associated with a significantly higher risk of an adverse outcome compared to NIPT in this risk-indicated cohort. The likelihood of miscarriage was significantly higher in the amniocentesis group (aOR: 1.91, 95% CI: 1.17–3.14, *p* = 0.025), as was the risk of IUD (aOR: 4.10, 95% CI: 2.05–8.20, *p* < 0.001). PTB risk was also increased (aOR: 1.96, 95% CI: 1.53–2.51, *p* < 0.001). LBW was significantly more prevalent in the amniocentesis group (aOR: 7.73, 95% CI: 5.40–11.05, *p* < 0.001), and the likelihood of delivering a SGA neonate was also increased (aOR: 1.45, 95% CI: 1.02–2.06, *p* = 0.040). A 1st-minute APGAR score < 7 was also more frequent in the amniocentesis group (aOR: 1.51, 95% CI: 1.06–2.16, *p* = 0.022), although the association with 5th-minute APGAR scores < 7 did not reach statistical significance (aOR: 1.45, 95% CI: 0.83–2.52, *p* = 0.193). Overall, the risk of composite maternal and perinatal adverse outcomes (aOR: 1.77, 95% CI: 1.41–2.22, *p* < 0.001) as well as composite fetal and neonatal adverse outcomes (aOR: 1.97, 95% CI: 1.50–2.58, *p* < 0.001) was significantly higher in the amniocentesis group compared to the NIPT group. No significant association was observed for PIHD, GDM, or ICP. **Conclusions**: Our findings showed that, apart from fetal loss, amniocentesis may be associated with adverse perinatal outcomes such as PTB, LBW, SGA and low APGAR scores.

## 1. Introduction

Prenatal diagnosis of fetal chromosomal abnormalities in pregnancies with increased risk factors provides families with essential information to prepare for birth and make informed decisions about the fetus’s care and future outcomes. Prenatal diagnostic tests also play a critical role in helping clinicians accurately diagnose diseases and plan appropriate treatment strategies. Most families want to be sure that a fetus at risk of genetic abnormalities is healthy. However, providing evidence-based information about the potential complications of invasive procedures is crucial to guide decision-making [1]. Amniocentesis is a widely used prenatal diagnostic procedure in which fetal cell samples are invasively obtained from the amniotic fluid to analyze the fetal genetic structure. It is the most commonly performed invasive prenatal diagnostic test and is recommended after the 15th week of gestation [2].

Despite its widespread use, the primary concern associated with amniocentesis is the risk of fetal loss [3]. However, there is still considerable variability in professional organizations’ recommendations regarding the risk of fetal loss associated with amniocentesis. There is also significant evidence to suggest that the risk of procedure-related fetal loss is much lower than currently stated by professionals [4,5,6,7]. Recent studies report procedure-related fetal loss rates ranging from 0.06% to 0.13%, reflecting improvements in technique and operator expertise [8,9]. Moreover, studies indicate that the risk of fetal loss may be even lower for other invasive diagnostic procedures, such as chorionic villus sampling (CVS) [10]. Due to conflicting results of studies, the procedure-related loss rate after invasive testing is controversial. The main reason for this is population differences and methodological heterogeneity in retrospective controlled studies. Additionally, research has identified several factors that may influence complication rates, including maternal age, gestational age, multiple needle insertions, operator experience, and the presence of fetal genetic or structural anomalies. The impact of these confounding factors on outcomes varies significantly between studies, contributing to the inconsistency in reported results [3,10].

In addition to fetal loss, there is limited but growing evidence regarding the long-term outcomes of prenatal diagnostic procedures. For example, data from the EuroPOP group demonstrated an increased prevalence of preterm birth in women who underwent amniocentesis [11]. Some studies have suggested that CVS may be associated with the subsequent development of preeclampsia [12,13]. Furthermore, operator experience has been implicated in adverse neonatal outcomes, such as low birth weight and fetal growth restriction, underscoring the importance of technical proficiency. The most common indications for diagnostic testing are first-trimester-screening-test positivity and advanced maternal age [14,15]. Additionally, advanced maternal age and first-trimester-screening-test serum markers have been shown to be associated with adverse pregnancy outcomes [15,16].

Over the last two decades, advancements in prenatal screening have significantly improved the detection and screening of fetal genetic conditions, particularly aneuploidies like Down syndrome. The primary objective of this study was to evaluate adverse pregnancy outcomes associated with amniocentesis, particularly fetal loss and preterm birth, using a cohort of women who underwent non-invasive prenatal testing (NIPT) with comparable clinical indications as a reference group. Secondary outcomes included neonatal parameters such as birth weight, fetal growth status, and APGAR scores. To ensure a more accurate and clinically relevant comparison, we selected a control group with comparable risk indications, addressing the limitation of previous studies that primarily used healthy pregnant women as controls. Given that variables such as population at risk, advanced maternal age and first-trimester screening test serum markers are independently associated with adverse pregnancy outcomes, it is also important to compare two groups with similar characteristics. This design allowed a comparison of outcomes between women undergoing amniocentesis and those opting for NIPT within a cohort with similar clinical indications, partially addressing potential confounding factors.

## 2. Materials and Methods

**Figure 1:**
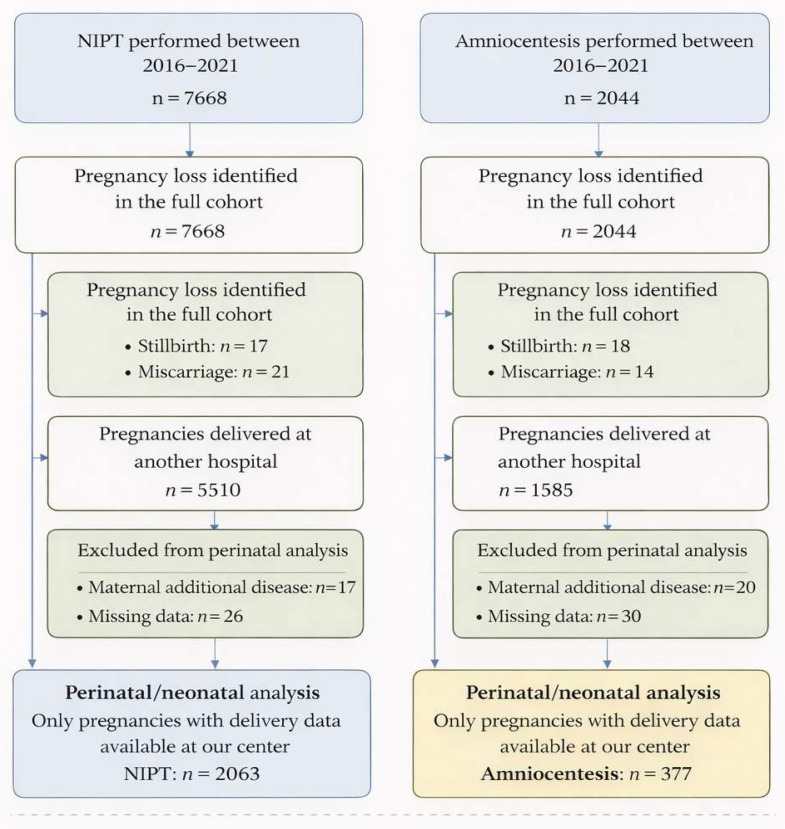
The flow chart of the study.

This retrospective cohort study was conducted using data collected between January 2016 and December 2021 at the University of Health Sciences Tepecik Training and Research Hospital, Turkey. The study received approval from the local ethics committee (approval no: 2022/01-12) and due to its retrospective nature, informed consent was waived with the approval of the local ethics committee. A total of 2044 pregnant women who underwent amniocentesis with reportable/normal results were included in the study. During the same period, 7668 pregnant women with reportable/normal NIPT results at our hospital served as the control group. Exclusion criteria included multiple pregnancies, fetal structural or genetic anomalies, suspected fetal infection, parental genetic abnormalities, maternal chronic diseases, and cases with missing data. Pregnancies delivered at other hospitals were not excluded from the overall cohort; however, perinatal and neonatal outcome analysis were restricted to cases with delivery data available at our center. Perinatal and neonatal outcomes were evaluated for 2063 pregnant women in the NIPT group and 377 pregnant women in the amniocentesis group whose birth data were available. The flow chart is shown in Figure 1.

Indications for amniocentesis included a high or moderate risk in first- and second-trimester screening tests (>1:1000), advanced maternal age (≥35 years), and the presence of positive ultrasonographic markers for aneuploidy. A local antiseptic was applied to the skin prior to the procedure, but no anesthesia was used. Amniocentesis was conducted after the 15th week of gestation using a 22-gauge (22 G) needle, inserted transabdominally under real-time ultrasound guidance. A total of 15–25 mL of amniotic fluid was aspirated. Ultrasound guidance was performed using a Samsung Ultrasound System HS70A (Samsung Medison Company, Seoul, Republic of Korea), equipped with an abdominal 4–8 MHz curvilinear transducer.

NIPT was performed free of charge within the scope of national health insurance at the Genetic Diagnostic Center of our hospital. NIPT was not used as a primary aneuploidy screening test. Indications for NIPT included a high or moderate risk in first- and second-trimester screening tests (>1:1000), advanced maternal age (≥35 years), and the presence of positive ultrasonographic markers for aneuploidy. NIPT was performed using next-generation sequencing (NGS) platforms, specifically NextSeq (Illumina, San Diego, CA, USA) or MiSeq (Illumina, USA). Cases with a fetal fraction below 2.8% were considered non-reportable by the manufacturer’s software algorithm and therefore excluded, as no valid NIPT result could be generated [17]. Both the amniocentesis and NIPT cohorts were derived from the same tertiary-care center and included women tested for identical predefined clinical indications (high or moderate screening risk, advanced maternal age ≥ 35 years, and/or positive ultrasonographic markers for aneuploidy). Thus, comparability was primarily addressed at the level of clinical indication.

Pregnancy outcomes of women who underwent NIPT and amniocentesis were examined from the hospital’s digital registration system. For pregnancy loss outcomes (miscarriage and intrauterine fetal demise), the entire cohort of women undergoing amniocentesis (*n* = 2044) and NIPT (*n* = 7668) was analyzed, as these events were captured through the institutional electronic registry regardless of the place of delivery. In contrast, detailed perinatal and neonatal outcomes—including gestational age at delivery, birth weight, fetal growth status, and APGAR scores—were available only for women who delivered at our center. Therefore, analysis of perinatal and neonatal outcomes were restricted to cases with complete delivery data (377 amniocentesis and 2063 NIPT cases). Adverse pregnancy outcomes included: miscarriage, intrauterine fetal demise (IUD), preterm birth (PTB), pregnancy-induced hypertensive diseases (PIHDs), gestational diabetes mellitus (GDM), intrahepatic cholestasis of pregnancy (ICP), and low birth weight (LBW), small for gestational age (SGA), low 1st and 5th minute APGAR scores (<7). Birth weight classifications were based on percentiles, with newborns below the 10th percentile classified as small for gestational age (SGA), those between the 10th and 90th percentiles as appropriate for gestational age (AGA), and those above the 90th percentile as large for gestational age (LGA). Newborns with a birth weight below 2500 g were classified as LBW, while those with a birth weight above 4000 g were classified as macrosomic. Miscarriage was defined as fetal loss before 24 weeks of gestation, while IUD was defined as fetal death at or beyond 24 weeks, prior to delivery. PIHD, encompassing gestational hypertension and preeclampsia, was defined according to the International Society for the Study of Hypertension in Pregnancy guidelines [18]. GDM was defined based on the 75 g oral glucose screening test according to International Association of Diabetes and Pregnancy Study Groups recommendations [19]. ICP was diagnosed by elevation in serum fasting bile acid level according to the Society for Maternal-Fetal Medicine criteria [20]. PTB was defined as delivery before 37 weeks of gestation and was further classified into subcategories: extremely preterm (<28 weeks), very preterm (28–32 weeks), moderately preterm (32–34 weeks), and late preterm (34–37 weeks). APGAR scores were evaluated separately at the 1st and 5th minutes. Newborns with APGAR scores < 7 at the 1st minute or at the 5th minute were considered to have low APGAR scores for the respective analysis. Composite maternal and perinatal adverse outcomes were defined as the presence of at least one of PTB, PIHD, GDM, or ICP. Composite fetal and neonatal adverse outcomes were defined as the presence of at least one of LBW, SGA, 1st minute APGAR scores < 7, or 5th minute APGAR scores < 7.

### Statistical Analysis

Statistical analysis were conducted using the Statistical Package for the Social Sciences (SPSS) version 26.0 (IBM Corporation, Armonk, NY, USA). A *p*-value of <0.05 was considered statistically significant. Student’s *t*-test was used to compare parametric data, with results presented as mean ± standard deviation (SD). Categorical variables were analyzed using the Chi-square test, with results reported as counts (*n*) and percentages (%). Logistic regression analysis were performed to identify independent predictors for adverse pregnancy outcomes, adjusting for potential confounding factors including maternal age and parity. Maternal age and parity were included in the multivariable model based on their significance in the univariate analysis and their established obstetric relevance as potential confounders. Logistic regression results are expressed as odds ratios (ORs), adjusted ORs (aORs), and 95% confidence intervals (CIs).

## 3. Results

Perinatal and neonatal outcomes were evaluated for 2063 pregnant women in the NIPT group and 377 pregnant women in the amniocentesis group whose birth data were available. The demographic and clinical characteristics of pregnant women in both groups are presented in Table 1. Mean maternal age, the proportion of women aged ≥35 years, and mean body mass index (BMI) were similar between the groups (*p* = 0.120, *p* = 0.372, and *p* = 0.420, respectively). However, multiparity was significantly more frequent in the amniocentesis group (82.2% vs. 73.4%, *p* < 0.001). Gestational age at delivery was significantly higher in the NIPT group (38.1 ± 2.8 weeks vs. 36.8 ± 3.1 weeks, *p* < 0.001). The cesarean section rate was significantly higher in the amniocentesis group (76.7% vs. 63.5%, *p* < 0.001). The mean birth weight was significantly lower in the amniocentesis group compared to the NIPT group (2913 ± 751 g vs. 3105.9 ± 644.4 g, *p* < 0.001) (Table 1).

The maternal and perinatal adverse outcomes of both groups are presented in Table 2. The rate of PTB was significantly higher in the amniocentesis group compared to the NIPT group (31.8% vs. 17.5%, *p* < 0.001). Additionally, the proportion of extremely preterm births (<28 weeks) was significantly increased among women who underwent amniocentesis (3.4% vs. 1.5%, *p* = 0.001). Furthermore, late preterm birth (34–37 weeks) was significantly more common in the amniocentesis group compared to the NIPT group (23.1% vs. 10.8%, *p* < 0.001). There was no significant difference in the incidence of PIHD between the groups (*p* = 0.287). Similarly, the incidence of GDM and ICP was comparable between the groups (*p* = 0.175 and *p* = 0.494, respectively). Composite maternal and perinatal adverse outcomes were significantly more frequent in the amniocentesis group compared to the NIPT group (38.2% vs. 26.5%, *p* < 0.001) (Table 2).

Univariate and multivariate analysis on independent predictors of maternal and perinatal adverse outcomes are presented in Table 3. Women who underwent amniocentesis had significantly higher odds of PTB compared to those undergoing NIPT (aOR: 1.96, 95% CI: 1.53–2.51, *p* < 0.001). Late preterm births (34–37 weeks) were also significantly more frequent in the amniocentesis group (aOR: 2.13, 95% CI: 1.60–2.84, *p* < 0.001). Although extremely preterm births (<28 weeks) were significantly associated with amniocentesis in the univariate analysis, this finding approached but did not reach statistical significance after adjusting for maternal age and parity (aOR: 1.96, 95% CI: 0.99–3.88, *p* = 0.053). Furthermore, composite maternal and perinatal adverse outcomes were significantly increased in the amniocentesis group compared with the NIPT group, even after adjusting for maternal age and parity (aOR: 1.77, 95% CI: 1.41–2.22, *p* < 0.001) (Table 3).

The comparison of pregnancy loss, fetal, and neonatal outcomes between the two groups are presented in Table 4. The analysis revealed that miscarriage rates were significantly higher in the amniocentesis group compared to the NIPT group (0.68% vs. 0.27%, *p* = 0.005). Similarly, the incidence of IUD was markedly increased in the amniocentesis group (0.88% vs. 0.22%, *p* < 0.001). LBW was significantly more common in pregnancies undergoing amniocentesis than in NIPT (23.6% vs. 2.8%, *p* < 0.001), while birth weights within the normal range (2500–4000 g) were less frequent (72.4% vs. 91.8%, *p* < 0.001). In terms of fetal growth patterns, SGA newborns were significantly more frequent in the amniocentesis group (14.3% vs. 10.1%, *p* = 0.014), while LGA newborns were more commonly observed in the NIPT group (11.9% vs. 16.5%, *p* = 0.024). Additionally, 1st minute APGAR scores < 7 were significantly more frequent in the amniocentesis group compared to the NIPT group (14.6% vs. 9.1%, *p* < 0.001). In parallel, 5th minute APGAR scores < 7 were significantly higher among newborns in the amniocentesis group (6.6% vs. 2.4%, *p* < 0.001). Composite fetal and neonatal adverse outcomes were significantly higher in the amniocentesis group compared to the NIPT group (32.3% vs. 17.3%, *p* < 0.001) (Table 4).

Univariate and multivariate analysis on independent predictors of fetal and neonatal adverse outcomes are presented in Table 5. The analysis demonstrated that the risk of miscarriage was significantly higher in the amniocentesis group compared to the NIPT group (aOR: 1.91, 95% CI: 1.17–3.14, *p* = 0.025). Additionally, the risk of IUD was substantially elevated in the amniocentesis group (aOR: 4.10, 95% CI: 2.05–8.20, *p* < 0.001). LBW was significantly more common in the amniocentesis group (aOR: 7.73, 95% CI: 5.40–11.05, *p* < 0.001), while birth weights within the normal range (2500–4000 g) were less frequent (aOR: 0.35, 95% CI: 0.26–0.46, *p* < 0.001). In terms of fetal growth patterns, the odds of being SGA were significantly higher in the amniocentesis group compared to NIPT (aOR: 1.45, 95% CI: 1.02–2.06, *p* = 0.040), while LGA outcomes were significantly lower (aOR: 0.60, 95% CI: 0.41–0.88, *p* = 0.009). Moreover, the risk of having a low 1st minute APGAR score (<7) was significantly increased in the amniocentesis group (aOR: 1.51, 95% CI: 1.06–2.16, *p* = 0.022). However, no significant association was found between the amniocentesis procedure and low 5th minute APGAR scores (<7) after adjustment (aOR: 1.45, 95% CI: 0.83–2.52, *p* = 0.193). Furthermore, composite fetal and neonatal adverse outcomes were significantly increased in the amniocentesis group compared with NIPT cases, independently of maternal age and parity (aOR: 1.97, 95% CI: 1.50–2.58, *p* < 0.001) (Table 5).

**Figure 2:**
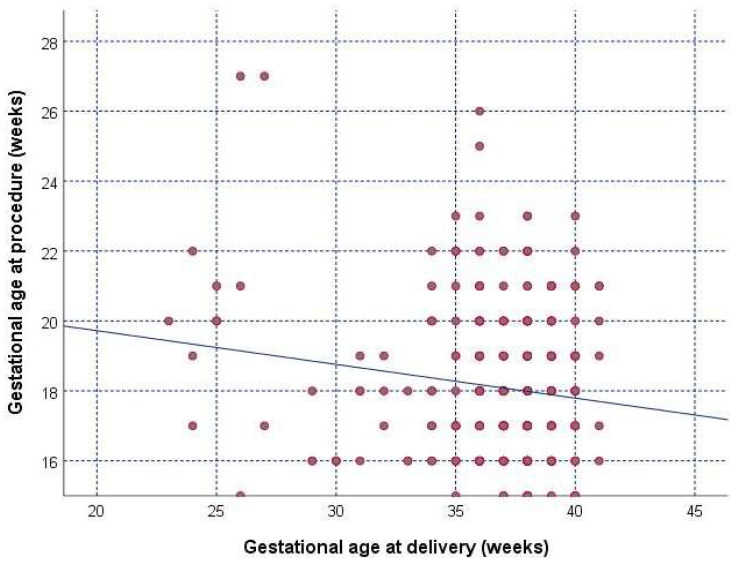
A weak inverse correlation was observed between the time of the procedure and the gestational age at delivery (r = −0.174, *p* = 0.001).

The correlation between gestational age at the procedure and gestational age at delivery in the amniocentesis group is shown in Figure 2. Accordingly, a weak inverse correlation was observed between the time of the procedure and the gestational age at delivery (r = −0.174, *p* = 0.001) (Figure 2).

## 4. Discussion

In this study, we comprehensively evaluated perinatal and neonatal outcomes in women who underwent amniocentesis, comparing them with those who underwent NIPT within a cohort of pregnancies with similar clinical indications and comparable baseline characteristics. Our findings demonstrated that the incidence of adverse outcomes, including miscarriage and IUD, was significantly higher in the amniocentesis group compared to the NIPT group. After adjusting for maternal age and parity, amniocentesis remained significantly associated with an increased risk of miscarriage and IUD. Additionally, amniocentesis was associated with an increased risk of PTB, particularly late preterm births (34–37 weeks). Although extremely preterm births (<28 weeks) initially showed a significant association in univariate analysis, this finding was not statistically significant after adjustment. Moreover, composite maternal and perinatal adverse outcomes, which include the presence of at least one of PTB, PIHD, GDM, or ICP, were significantly more frequent in the amniocentesis group compared to the NIPT group. Regarding neonatal outcomes, LBW and SGA were significantly more common in the amniocentesis group. Furthermore, low 1st minute APGAR scores (<7) were significantly more frequent in the amniocentesis group. However, there was no significant association between amniocentesis and low 5th minute APGAR scores (<7) after adjustment. Composite fetal and neonatal adverse outcomes, defined as the presence of at least one of LBW, SGA, or low 1st and 5th minute APGAR scores, were significantly increased in the amniocentesis group even after adjusting for confounders. Importantly, no significant association was identified between amniocentesis and maternal complications such as PIHD, GDM, or ICP. These findings suggest that, while amniocentesis remains an indispensable diagnostic tool in prenatal care, it may be associated with an increased risk of adverse perinatal and neonatal outcomes compared to NIPT. Therefore, it is essential for healthcare providers to carefully assess the risks and benefits, provide thorough patient counseling, and consider alternative screening options when appropriate.

Amniocentesis is the most commonly performed invasive procedure in prenatal diagnosis, with pregnancy loss being its most serious complication. Previous studies have reported procedure-related fetal loss rates ranging from 0.19% to 1.53% [10]. However, many of these studies included heterogeneous populations, which may have influenced the reported complication rates. The first prospective randomized study by Tabor et al. estimated the procedure-related fetal loss rate at 1% [21]. Twenty-two years later, the same author published data reporting a fetal loss rate of 0.5% from 32,852 women who underwent amniocentesis [22]. In this study, the authors used a control group consisting of women who did not undergo amniocentesis to establish the background risk of fetal loss. However, this control group was not matched for key risk factors, such as advanced maternal age and first-trimester screening serum markers, which may have influenced the reported outcomes. The study also indicated that gestational age at the time of the procedure and the level of institutional expertise might impact complication rates. Further studies conducted by Backer et al., which included unmatched control groups, reported fetal loss rates ranging from 0.17% to 0.52%, with a trend toward lower risk as operator experience increased [23]. Similarly, Beta et al. estimated the procedure-related fetal loss rate at 0.36% and identified several risk factors associated with miscarriage, including advanced maternal age, maternal weight and height, race, assisted reproductive technology, chronic hypertension, and low pregnancy-associated plasma protein-A (PAPP-A) multiples of the median (MoM ≤ 0.3) [24]. In our study, we evaluated procedure-related fetal loss rates in patient groups with similar risk factors. We specifically focused on evaluating procedure-related fetal loss rates in patient groups with comparable clinical indications, addressing a critical gap in the existing literature. The observed fetal loss rate in our cohort was 0.68% before 24 weeks and 0.88% after 24 weeks of gestation. The aOR for miscarriage and IUD was significantly higher in the amniocentesis group compared to the NIPT group, indicating an increased risk even after adjusting for maternal age and parity. Our findings suggest that the risk of fetal loss associated with amniocentesis remains significantly higher than in a similar risk population, even after adjusting for confounding factors. This underscores the importance of careful patient selection, thorough counseling, and meticulous procedural techniques to minimize risks.

There were few studies evaluating perinatal outcomes following amniocentesis and the existing literature presents conflicting results. In the first study to evaluate the risk of PTB after amniocentesis, Tongsong et al. found no increased risk of procedure-related PTB [25]. Similarly, in a randomized controlled trial involving 4606 women, Tabor et al. also reported no significant association between amniocentesis and PTB [22]. In contrast, Theodora et al. observed a higher incidence of PTB in women who underwent amniocentesis compared to the control group, though this difference did not reach statistical significance [26]. On the other hand, Sant-Cassia et al. reported a higher rate of PTB after amniocentesis [27]. A large multicenter study conducted by the EuroPOP group further supported this association, showing that amniocentesis was associated with an increased risk of PTB. The authors hypothesized that amniocentesis may lead to PTB, especially due to a chronic inflammatory response associated with membrane perforation and an increase in metalloproteinase and prostaglandin levels, which are responsible for the onset of labor [11]. Consistent with these findings, our study showed that the amniocentesis procedure was associated with an increased risk of PTB. We found that the adjusted analysis revealed that women undergoing amniocentesis had significantly higher aORs of PTB and late preterm birth compared to those undergoing NIPT. However, extremely preterm birth did not reach significance when confounders were added. Composite maternal and perinatal adverse outcomes were significantly higher in the amniocentesis group compared with NIPT cases, independently of maternal age and parity. These findings suggest that while extremely preterm births might not be directly influenced by the procedure after adjusting for confounding factors, the risk of late PTB remains significant. Moreover, we observed a statistically significant but weak inverse correlation between gestational age at amniocentesis and gestational age at delivery. This finding should be interpreted cautiously and does not imply a causal effect of procedure timing, because the gestational age at amniocentesis may reflect underlying clinical indications and risk severity [28,29]. Previous studies have suggested that amnio-chorionic separation, delayed membrane healing responses, and elevated inflammatory mediators may contribute to this effect [28,30].

In our study, we also evaluated neonatal outcomes and found that the incidence of LBW, SGA, and low 1st APGAR scores (<7) was significantly higher in pregnancies that underwent amniocentesis compared to those undergoing NIPT. No significant association was found between amniocentesis and low 5th minute APGAR scores (<7). Composite fetal and neonatal adverse outcomes were significantly higher in the amniocentesis group compared with NIPT cases, independently of maternal age and parity. Limited studies have examined neonatal outcomes of pregnancies that underwent amniocentesis. Previous research has indicated an association between PTB and adverse neonatal outcomes such as LBW and lower APGAR scores [31]. However, Wisetmongkolchai et al. investigated the impact of operator experience on amniocentesis outcomes and found that LBW and fetal growth restriction were significantly more common when the procedure was performed by less-experienced operators [32]. The authors suggested that this increased risk could be attributed to placental damage, potentially caused by multiple needle insertions, prolonged procedure times, and the presence of bloody amniotic fluid in procedures conducted by non-expert operators. Due to limitations in our dataset, we were unable to establish a direct correlation between operator experience and potential procedure-related complications. Additionally, our study did not include data on multiple needle insertions, placental needle passage, or amniotic fluid content, which may contribute to neonatal risks. Despite these limitations, our findings suggest that amniocentesis may be associated with adverse neonatal outcomes beyond fetal loss, emphasizing the need for further research to better understand these potential risks and their underlying mechanisms.

Our study also investigated the potential association between amniocentesis and PIHD. Contrary to our initial hypothesis, we found no significant association between the amniocentesis procedure and the occurrence of PIHD, even after adjusting for maternal age and parity. Previous studies have primarily examined the association between prenatal diagnostic procedures and PIHD, with a particular focus on CVS. To our knowledge, no study evaluating the association of the amniocentesis procedure with PIHD has been published yet. Studies evaluating the association between CVS and PIHD presented conflicting results. Maruotti et al. and Odibo et al. suggested that the risk of PIHD was less in cases undergoing CVS [33,34]. On the other hand, other studies have suggested that CVS may actually increase the risk of PIHD [35,36,37]. The authors attributed this association to early damage to the placenta that may occur due to the procedure. As outlined in clinical guidelines, there is a theoretical concern that needle passage through the placenta during amniocentesis might increase the risk of complications, including hypertensive disorders [2]. Despite this concern, our data did not demonstrate a statistically significant increase in PIHD rates among women who underwent amniocentesis. Therefore, larger-scale prospective studies are needed to further investigate the potential relationship between amniocentesis and PIHD, incorporating additional factors such as procedure duration, placental location, and needle entry localization to better assess its impact on maternal and fetal outcomes.

We also evaluated the potential association between amniocentesis and metabolic conditions, including GDM and ICP, and found no association between amniocentesis and these conditions. While the pathophysiological mechanisms underlying these conditions differ from those related to hypertensive disorders, both GDM and ICP are influenced by inflammatory and vascular changes during pregnancy. It has been suggested that inflammatory responses during pregnancy, including cytokine production and alterations in vascular function, may contribute to the development of these maternal complications [38,39,40]. However, our data did not support this hypothesis, indicating that the potential inflammatory impact of the procedure may not be sufficient to influence the development of GDM or ICP. Our findings are consistent with limited evidence suggesting that maternal metabolic and hepatic complications may be more influenced by pre-existing maternal factors rather than by the amniocentesis procedure itself [41,42].

This study had several limitations that should be acknowledged when interpreting the results. The most significant limitation was its retrospective design, which inherently restricts quality of information available in patient records. Although both cohorts were selected based on identical predefined clinical indications, residual heterogeneity in screening-risk severity cannot be excluded, as detailed quantitative screening metrics (e.g., combined-risk estimates and serum marker values) were not consistently available. Therefore, the possibility of residual confounding should be considered when interpreting the associations observed. Additionally, NIPT cases with fetal fraction below the laboratory reporting threshold were classified as non-reportable by the manufacturer’s software algorithm and therefore could not be included in the NIPT cohort. Although low fetal fraction has been associated with adverse pregnancy outcomes in previous studies, this exclusion may have influenced baseline risk distribution between groups. Furthermore, perinatal outcomes of women who did not deliver at our center could not be assessed in either the case or control groups, potentially leading to incomplete outcome data. Important procedure-related details, such as the number of needle entries, placental placement, and operator experience, were not available for analysis and may have influenced the observed outcomes. This study also had several strengths. A key strength was the use of a control group consisting of pregnancies with similar risk factors who underwent NIPT. This design allowed for a more balanced comparison between groups with similar clinical indications. However, residual confounding cannot be completely excluded due to the retrospective design and the absence of detailed data on several potential confounders. Additionally, pregnancies with structural and genetic anomalies were excluded, reducing potential confounding effects. Moreover, this study is among the few studies specifically evaluating perinatal outcomes following amniocentesis, contributing valuable insights to the existing literature.

## 5. Conclusions

In conclusion, our study found that the risk of miscarriage and IUD following amniocentesis was significantly higher compared to the NIPT group. Additionally, our findings suggest that amniocentesis may be associated with adverse perinatal outcomes, including PTB, LBW, SGA, and low APGAR scores. Furthermore, composite maternal and perinatal adverse outcomes, as well as composite fetal and neonatal adverse outcomes, were significantly more frequent in the amniocentesis group. These results underscore the critical importance of careful risk assessment and thorough patient counseling prior to the procedure, particularly when alternative non-invasive prenatal screening options are available. Despite the comparative analysis presented in this study, further prospective, large-scale investigations are warranted to assess the long-term pregnancy and neonatal outcomes associated with amniocentesis. Future research should also explore potential mitigating strategies—such as optimizing procedural techniques, enhancing operator experience, and implementing supportive care protocols—to minimize the associated risks and improve patient safety.

## Figures and Tables

**Table 1:** Demographic and clinical characteristics of the study population.

	Non-Invasive Prenatal Test *n* = 2063	Amniocentesis *n* = 377	*p*
**Maternal age (years)**	32 ± 6.5	31 ± 7	0.120
**≥35 years**	515 (25%)	86 (22.9%)	0.372
**BMI (kg/m^2^)**	26.6 ± 5.1	26.3 ± 4.5	0.420
**Parity**			<0.001
**Nulliparous**	548 (26.6%)	67 (17.8%)	
**Multiparous**	1515 (73.4%)	310 (82.2%)	
**Gestational age at delivery (weeks)**	38.1 ± 2.8	36.8 ± 3.1	<0.001
**Delivery type**			<0.001
**Vaginal delivery**	753 (36.5%)	88 (23.3%)	
**Cesarean section**	1310 (63.5%)	289 (76.7%)	
**Birth weight (g)**	3105.9 ± 644.4	2913 ± 751	<0.001

BMI: Body mass index.

**Table 2:** Comparison of maternal and perinatal adverse outcomes.

		Non-Invasive Prenatal Test *n* = 2063		Amniocentesis *n* = 377		*p*
		*n*	%	*n*	%	
**PTB**	**No**	1702	82.5%	257	68.2%	<0.001
	**Yes**	361	17.5%	120	31.8%	
**Extremely preterm (<28 weeks)**	**No**	2031	98.5%	364	96.6%	0.001
	**Yes**	32	1.5%	13	3.4%	
**Very preterm (28–32 weeks)**	**No**	2011	97.5%	364	96.6%	0.303
	**Yes**	52	2.5%	13	3.4%	
**Moderately preterm (32–34 weeks)**	**No**	2008	97.4%	370	98.1%	0.358
	**Yes**	55	2.6%	7	1.9%	
**Late preterm (34–37 weeks)**	**No**	1841	89.2%	290	76.9%	<0.001
	**Yes**	222	10.8%	87	23.1%	
**PIHD**	**No**	1851	89.8%	345	91.5%	0.287
	**Yes**	212	10.2%	32	8.5%	
**GDM**	**No**	1939	94%	361	95.8%	0.175
	**Yes**	124	6%	16	4.2%	
**ICP**	**No**	2052	99.5%	376	99.7%	0.494
	**Yes**	11	0.5%	1	0.3%	
Composite maternal and perinatal adverse outcomes *	**No**	1516	73.5%	233	61.8%	<0.001
	**Yes**	547	26.5%	144	38.2%	

PTB: Preterm birth; PIHDs: Pregnancy-induced hypertensive diseases; GDM: Gestational diabetes mellitus; ICP: Intrahepatic cholestasis of pregnancy. * Composite maternal and perinatal adverse outcomes are defined as the presence of at least one of the following adverse outcomes: Preterm birth, pregnancy-induced hypertensive diseases, gestational diabetes mellitus, or intrahepatic cholestasis of pregnancy.

**Table 3:** Univariate and multivariate analysis on independent predictors of maternal and perinatal adverse outcomes.

	Univariate			Multivariate *		
	OR	95% CI	*p*	aOR	95% CI	*p*
**PTB**	2.20	1.72–2.81	<0.001	1.96	1.53–2.51	<0.001
**Extremely preterm (<28 weeks)**	2.26	1.17–4.36	0.001	1.96	0.99–3.88	0.053
**Late preterm (34–37 weeks)**	2.31	1.75–3.04	<0.001	2.13	1.60–2.84	<0.001
Composite maternal and perinatal adverse outcomes **	1.71	1.36–2.15	<0.001	1.77	1.41–2.22	<0.001

OR: Odds ratio; aOR: Adjusted odds ratio; CI: Confidence interval; PTB: Preterm birth. * Multivariate analysis adjusted for maternal age and parity. ** Composite maternal and perinatal adverse outcomes are defined as the presence of at least one of the following adverse outcomes: Preterm birth, pregnancy-induced hypertensive diseases, gestational diabetes mellitus, or intrahepatic cholestasis of pregnancy.

**Table 4:** Comparison of pregnancy loss, fetal, and neonatal outcomes.

		Non-Invasive Prenatal Test *n* = 2063		Amniocentesis *n* = 377		*p*
		*n*	%	*n*	%	
No *		7630	99.51%	2012	98.44%	<0.001
Miscarriage *		21	0.27%	14	0.68%	0.005
Intrauterine fetal demise *		17	0.22%	18	0.88%	<0.001
**LBW (<2500 g)**	**No**	2005	97.2%	288	76.4%	<0.001
	**Yes**	58	2.8%	89	23.6%	
**2500–4000 g**	**No**	170	8.2%	104	27.6%	<0.001
	**Yes**	1893	91.8%	273	72.4%	
**Macrosomia (≥4000 g)**	**No**	1951	94.6%	362	96%	0.243
	**Yes**	112	5.4%	15	4%	
**SGA**	**No**	1855	89.9%	323	85.7%	0.014
	**Yes**	208	10.1%	54	14.3%	
**AGA**	**No**	549	26.6%	99	26.3%	0.886
	**Yes**	1514	73.4%	278	73.7%	
**LGA**	**No**	1722	83.5%	332	88.1%	0.024
	**Yes**	341	16.5%	45	11.9%	
**1st minute APGAR scores < 7**	**No**	1876	90.9%	322	85.4%	<0.001
	**Yes**	187	9.1%	55	14.6%	
**5th minute APGAR scores < 7**	**No**	2014	97.6%	352	93.4%	<0.001
	**Yes**	49	2.4%	25	6.6%	
Composite fetal and neonatal adverse outcomes **	**No**	1705	82.7%	259	68.7%	<0.001
	**Yes**	358	17.3%	118	31.3%	

LBW: Low birth weight; SGA: Small for gestational age; AGA: Appropriate for gestational age; LGA: Large for gestational age. * Miscarriage and intrauterine fetal demise analysis were performed in the full cohort (NIPT *n* = 7668; amniocentesis *n* = 2044). ** Composite fetal and neonatal adverse outcomes are defined as the presence of at least one of the following adverse outcomes: Low birth weight, small for gestational age, 1st minute APGAR scores < 7, or 5th minute APGAR scores < 7.

**Table 5:** Univariate and multivariate analysis on independent predictors of fetal and neonatal adverse outcomes.

	Univariate			Multivariate *		
	OR	95% CI	*p*	aOR	95% CI	*p*
Miscarriage **	2.51	1.27–4.94	0.005	1.91	1.17–3.14	0.025
Intrauterine fetal demise **	4.00	2.05–7.77	<0.001	4.10	2.05–8.20	<0.001
**LBW (<2500 g)**	7.78	5.61–10.68	<0.001	7.73	5.40–11.05	<0.001
**2500–4000 g**	0.23	0.17–0.31	<0.001	0.35	0.26–0.46	<0.001
**SGA**	1.49	1.08–2.05	0.014	1.45	1.02–2.06	0.040
**LGA**	0.68	0.49–0.95	0.024	0.60	0.41–0.88	0.009
**1st minute APGAR scores < 7**	1.71	1.24–2.36	<0.001	1.51	1.06–2.16	0.022
**5th minute APGAR scores < 7**	2.91	1.77–4.78	<0.001	1.45	0.83–2.52	0.193
Composite fetal and neonatal adverse outcomes ***	2.17	1.69–2.77	<0.001	1.97	1.50–2.58	<0.001

OR: Odds ratio; aOR: Adjusted odds ratio; CI: Confidence interval; LBW: Low birth weight; SGA: Small for gestational age; LGA: Large for gestational age. * Multivariate analysis adjusted for maternal age and parity. ** Miscarriage and intrauterine fetal demise analysis were performed in the full cohort (NIPT *n* = 7668; amniocentesis *n* = 2044). *** Composite fetal and neonatal adverse outcomes are defined as the presence of at least one of the following adverse outcomes: Low birth weight, small for gestational age, 1st minute APGAR scores < 7, or 5th minute APGAR scores < 7.

## Data Availability

The data presented in this study are available on request from the corresponding author due to a reasonable reason, with patient names anonymized.
